# Identification and Functional Distribution of Intracellular Ca^2+^ Channels in Mouse Lacrimal Gland Acinar Cells

**DOI:** 10.2174/1874364100701010008

**Published:** 2007-12-04

**Authors:** W.E Medina-Ortiz, E.V Gregg, A.M Brun-Zinkernagel, P Koulen

**Affiliations:** Department of Pharmacology and Neuroscience, University of North Texas Health Science Center, Fort Worth, TX 76107, USA

## Abstract

We have determined the presence and cellular distribution of intracellular calcium channels, inositol 1, 4, 5-trisphosphate receptors (IP3Rs) and ryanodine receptors (RyRs) in adult and postnatal (P10) lacrimal gland acinar cells. Western blot analysis of both P10 cultures and adult tissue identified the presence of each IP_3_R and RyR isotypes. The immunocytochemistry analysis showed a differential cellular distribution of these calcium channels where the nuclear envelope, endoplasmic reticulum (ER) and Golgi apparatus membranes represent areas with highest levels of channel expression. This IP_3_R and RyR isotype distribution is confirmed by the immuno-EM results. The findings described in this study are in agreement with published pharmacological data that shows the participation of these channels in the secretion process of the lacrimal gland acinar cells. Furthermore, the differential subcellular distribution between the isoforms could indicate a potential role of these intracellular Ca^2+ ^channels on the regulation of specific cellular functions.

## INTRODUCTION

The lacrimal gland plays a major role in the secretion and production of tear fluid components essential for eye maintenance and function. Absent or inadequate tear fluid secretion by lacrimal acinar cells can be the consequence of cell stress, infection, or cell death [1]. Damage or dysfunction of the lacrimal gland’s capacity for secretion can eventually create disturbances of the ocular surfaces including immunological syndromes including dry eye disease, secondary infections, or Sjögren’s syndrome [2, 3]. In view of that, it is important to understand the secretion process and the key elements required in the lacrimal gland.

Tear secretion of the lacrimal gland cells is mainly in response to both cholinergic and adrenergic stimuli and results from controlled changes in cytosolic Ca^2+ ^concentration [4, 5]. Studies on rodent lacrimal acinar cells have identified pathways involved in the secretory function. One important factor is the second messenger inositol 1,4,5-trisphosphate (IP_3_) and its derivatives. IP_3_ functions by releasing Ca^2+^ into the cytosol from intracellular stores, especially after a cholinergic stimulus [6-9]. IP_3 _is capable of releasing Ca^2+^ from intracellular stores by binding to and activating its receptor in the endoplasmic reticulum (ER) membrane, the IP_3_ receptors (IP_3_Rs). IP_3_Rs are members of a family of intracellular Ca^2+^ channels. There are three known subtypes and they can form both homo- and hetero-tetrameric complexes. These complexes work as ligand–gated ion channels and are regulated by both IP_3_ and Ca^2+^ [10, 11]. The second major group of intracellular Ca^2+^ channels is formed by the ryanodine receptors (RyRs) [12,  13]. Originally isolated from mammalian muscle cells and identified by their ability to bind to the plant alkaloid ryanodine, RyRs have now been identified in different organisms and tissues [13]. There are three known RyR isoforms, RyR1, RyR2 and RyR3, and they form tetrameric ion channels. These receptors share high sequence similarity with IP_3_R, particularly in the Ca^2+^ channel pore regions. Similar to IP_3_Rs, RyRs are regulated by the intracellular Ca^2+^ concentration and are therefore also called Ca^2+^-dependent Ca^2+^ release channels [12, 13]. RyR can also be co-regulated by the second messenger cADP-ribose [14].

Little is known about RyR expression and function in lacrimal glands but there are a few reports that show evidence for the possible involvement of RyR in the intracellular signaling pathways of secretory acinar cells in rat lacrimal gland [14, 15] and in other organs with homologous cell types such as pancreatic acinar cells [16, 17]. When rat lacrimal gland acinar cells are stimulated through the cholinergic pathway, IP_3_Rs but not RyRs are involved in Ca^2+^ transients [15]. However, under adrenergic stimulation lacrimal acinar cell function is IP_3 _independent and pharmacological evidence suggests that predominantly RyRs are involved in the β-adrenergic stimulation of lacrimal acinar cells *via *cADP-ribose [15].

Despite the pharmacological evidence for the involvement of IP_3_R and RyR in the release of Ca^2+^ from intracellular stores in lacrimal acinar cells [15, 18, 19], the identification of isoforms and their distribution is still lacking. Therefore, the goal of the present study was to identify and determine the cellular distribution of intracellular calcium channels in adult mouse lacrimal gland tissue and in postnatal primary acinar cell cultures. Findings of these immunochemical analyses will provide the cytological and molecular biological basis with respect to IP_3_R and RyR expression and distribution in the lacrimal acinar cells needed to investigate their contribution to the secretory function and isoform specific pharmacological properties.

## MATERIALS AND METHODS

All experiments described in the present study were carried out in accordance with the National Institutes of Health, UNTHSC guidelines for the welfare, care and use of experimental animals and the ARVO Statement for the Use of Animals in Ophthalmic and Vision Research.

### Culture Medium and Materials

Dulbecco’s minimium essential medium without sodium pyruvate (DMEM) (Hyclone, Logan, UT), 100X penicillin-streptomycin (p/s, Cellgro, Herndon, VA), heat inactivated bovine growth serum (BGS) (Hyclone, Logan, UT), Mouse laminin (Sigma, St. Louis, MO), soybean trypsin inhibitor (STI)_(Sigma, St. Louis, MO), EGTA (Sigma, St. Louis, MO), Collagenase blend (Sigma, St. Louis, MO), 0.25% trypsin (Hyclone, Logan, UT), and bovine serum albumin, fraction V (Sigma, St. Louis, MO) were used in isolating, culturing, and for glass coverslips.

### Western Blot Materials

RIPA buffer with protease inhibitors (Santa Cruz, Santa Cruz, CA), Dual Color Molecular size ladder (Bio-Rad, Hercules, CA), Chemo ladder (Pierce, Rockford, IL), Tween-20 (Pierce, Rockford, IL) Methanol (Fisher, Fair Lawn, NJ), 5% Tris-HCl gel (Bio-Rad, Hercules, CA), PVDF membranes (PALL Life Sciences, Ann Arbor, MI) and Super Signal West Dura Extended Substrate/Secondary HRP-conjugated antibodies Kit (Pierce, Rockford, IL) were materials used for Western blot work.

### Materials for Immunostaining

Paraformaldehyde (PFA) (Fisher, Fair Lawn, NJ), 0.1M PBS pH 7.4 (Fisher, Fair Lawn, NJ), Triton-X 100 (MP Biomedicals, Aurora, OH), -Prolong with DAPI (Molecular Probes, Carlsbad, CA), Aqua-Polymount (Polysciences, Inc., Warrington, PA), O.C.T. Compound (Sakura Finetek, Torrence, CA), were used for fluorescent microscopy. For electron microscopy, the following materials were used: para-formaldehyde (Electron Microscopy Sciences [EMS], Ft. Washington, PA), glutaraldehyde (Polysciences, Inc., Warrington, PA), Lowicryl K4M (Electron Microscopy Sciences, Ft. Washington, PA), benzoin ethylether (Electron Microscopy Sciences, Ft. Washington, PA), Glycine (Sigma, St. Louis, MO), Bovine Serum Albumin (Sigma, St. Louis, MO), normal serum (Vector Laboratories Inc., Burlingame, CA), Aurion Immuno Gold Reagent (Electron Microscopy Sciences, Ft. Washington, PA), and Uranyl Acetate (Polysciences, Inc., Warrington, PA).

### Antibodies

Rabbit polyclonal antibodies raised against RyR1, 2 and 3 (AB9078, AB9080, and AB9082 respectively; Chemicon, Temecula, CA) and IP_3_R1, 2 and 3 and (AB9072, AB9074, and AB9076, respectively; Chemicon, Temecula, CA) were used at 1:1000 for immunocytochemistry, immunohistochemistry, and Western blotting and antibodies against IP_3_R type 1 #407144 (Calbiochem, San Diego, CA; used at 1:2000 for Western Blot), and IP_3_R type 3 #I7529 (Sigma, St. Louis, MO; used at 1:1500 for Western Blot). Mouse monoclonal antibodies against RyR1 (clone 34C; MA3-925, Affinity BioReagents, Golden, CO; concentrated supernatant in a 1:100 dilution for immunocytochemistry and Western blot) and RyR2 (clone C3-33; MA3-916, Affinity BioReagents, Golden, CO; 1:200 dilution for immunocytochemistry and Western blot) were used and produced identical results. Secondary goat anti-rabbit or mouse IgG antibodies coupled to Alexa Fluor^®^ 594 (Molecular Probes, Carlsbad, CA) were used for immunofluorescence detection.

### Isolation of Adult Acinar Cells of the Lacrimal Gland

Adult Swiss-Webster mice were euthanized by CO_2_ overexposure and lacrimal glands were dissected and cut into pieces of approximately 1-2 mm^3 ^size for enzymatic dissociation (at 37^o^C). Tissue was incubated for 10 min in 0.25% trypsin which was removed followed by a 5 min wash with STI (0.2 mg/ml in 0.5% BSA-2.5 mM EGTA/DMEM without Na pyruvate). Pieces of gland were subsequently incubated with collagenase solution (0.4 mg/ml in 0.5% BSA/DMEM without Na pyruvate) for 10 min and mechanically dissociated by trituration with a sterile, fire-polished glass Pasteur pipette. Collagenase solution was removed following centrifugation and the cell pellet was washed with media (10% BGS, 1% p/s in DMEM, 37^o^C) to remove any remaining enzyme. Cells were resuspended in 10 ml media and cells were plated on 10 µg/ml laminin coated glass coverslips. Cells were allowed to attach for 2 hours in a humified atmosphere of 95% air, 5% CO_2_ at 37^o^C.

### Postnatal Lacrimal Gland Primary Cultures

Isolation of acinar cells from postnatal days 10-11 C57BL/6J mice was performed as described for adult acinar cells under sterile conditions. After seeding of cells on laminin coated glass coverslips, cells were allowed to attach for 2 hours at 37^o^C, with humidity and 5% CO_2_. Subsequently, 5% CO_2 _conditioned medium was added and cultures were maintained for 4-14 days *in vitro* (DIV) in a incubator with a humidified atmosphere of 95% air and 5% CO_2 _at 37^o^C. For Western blot analysis, cells were cultured in standard cell culture plastic flasks for 10-14 days.

## IMMUNOSTAINING

### Isolated and Cultured Isolated Cells

Attached cells were fixed for 20 min in 4% PFA (0.01 M PBS, pH 7.4) at room temperature (RT) and fixative was removed with two 10 min washes using PBS (0.01 M PBS, pH 7.4). Immunostaining consisted of blocking the cells for an hour at RT with blocking solution (10% normal goat serum, 1% BSA and 0.05% Triton-X 100 in 0.01 M PBS) and incubated overnight with the corresponding primary antibody dilution (antibodies dilution solution is 3% normal goat serum, 1% BSA and 0.05% Triton-X 100 in 0.01 M PBS) in a humidified chamber, protected from light and at 4^o^C. After 3 PBS washes (0.01 M), cells were incubated with secondary antibody conjugated to Alexa Fluor^®^ 594 for an hour at RT, in a humidified chamber and protected from light. After washing, coverslips were mounted on slides using Prolong with DAPI mounting solution (Molecular Probes, Carlsbad CA). Negative controls consisted of the omission of primary antibody from the incubation steps.

### Tissue Cryosections

Adult lacrimal glands were harvested from CO_2_-euthanized adult mice and fixed with 4% PFA in PBS (0.01 M) for 30 minutes, then cryoprotected with a serial saturation with 10, 20, and 30% sucrose in PBS (0.01 M) and stored at -20^o^C in the 30% sucrose solution until needed. For cryosectioning, thawed tissue was placed in OCT Compound (Sakura Finetek U.S.A., Torrance, CA) for 30 minutes at room temperature, frozen and then sectioned at 12 µm thickness. The sections were placed on silane coated slides (Mercedes Medical, Sarasota, FL) and were then stored at –20ºC until used in immunocytochemistry experiments. Immunostaining was performed as previously described [20-22].

### Imaging Analysis

Images acquired using an Olympus IX70 epifluorescence microscope with SimplePCI version 5.3.1 image acquisition and analysis software (Compix Inc., Hamamatsu Photonics Management, Sewickley, PA).

### Western Blot Analysis

Lacrimal gland tissue from adult mice or cultured lacrimal acinar cells after 11-14 DIV were homogenized with RIPA buffer containing 250 mM sucrose, 5 mM Hepes, 100 mM EGTA, and a mixture of protease inhibitors (10 ug/ml trypsin, 1mM pepstatin, 10 mM leupeptin and 2 mg/ml aprotonin). Total protein homogenate (30-50 ug) was electrophoretically separated for 2 hours in a 5% Tris-HCl SDS-PAGE. Following electrophoresis, gels were equilibrated and proteins transferred for 4 hours to a PVDF membrane. Membranes were blocked with blocking solution (5% BSA in washing solution) for an hour at RT. After that, membranes were incubated overnight with the corresponding diluted primary antibody at 4^o^C. Then, membranes were washed in washing solution and incubated with the appropriate HRP-conjugated secondary antibody for 1h (1:2000), at room temperature. Immunoreactive bands were detected with cheminoluminescence by the addition of Super Signal West Dura Substrate (Pierce, Rockford, IL) and images were captured using a CCD camera as part of a UVP Biomaging System with EpiChemi3 Darkroom and LabWorks 4.5 (UVP, Inc., Upland, CA).

### EM Analysis

Mouse lacrimal glands were immersion fixed in PBS containing 4% paraformaldehyde and 0.5% glutaraldehyde for 2-5 hours at 4^o^C. After rinsing in PBS, the tissue was dehydrated in a graded series of ethanol (10, 30, 50, 70, 95, and 3 x 100%) followed by infiltration with Lowicryl K4M (Electron Microscopy Sciences [EMS], Ft. Washington, PA) according to the manufacturer’s recommendation (except that all steps were performed at 4^o^C and the initiator C was replaced by the same amount of benzoin ethylether). The blocks were UV polymerized starting at 4^o^C, and slowly raising the temperature to 16^o^C over 24 hours. UV polymerization was then continued at room temperature for a total of 3-5 days. The tissue blocks were sectioned on an ultramicrotome (Reichert Ultracut S) at 70-100 nm thick thickness and were collected onto 200 mesh Formvar/Carbon coated grids. The immunostaining was performed on parafilm by putting the grids section side down onto the solution droplets. The grids were transferred from drop to drop by means of an anti-capillary forceps. The forceps was cleaned in 100% ethanol after each antibody. To quench free aldehydes, the sections were first incubated 3 times (5 min each) with 0.05 M glycine in 0.01M PBS. To block nonspecific antibody binding, the sections were then incubated for 15 min in incubation buffer [PBS (10 mM phosphate buffer, 150 mM NaCl) pH 7.4, 1% bovine serum albumin] containing 5% normal serum from the secondary antibody host animal (e.g. goat serum). The incubation times for the primary antibody as well as for the gold coupled secondary antibody (Electron Microscopy Sciences, Ft. Washington, PA; Aurion Immuno Gold Reagent, 15nm goat anti-rabbit or goat anti-mouse, 1:30) were 1 hour each. The grids were rinsed 6 times (5 minutes each rinse) between each incubation step. After the gold-complex incubation, grids were rinsed 3 times in the incubation buffer (5 min each), fixed in 1-2% glutaraldehyde in PBS for 15-20 min, rinsed 2 times (5 min each) in PBS, further rinsed in distilled water 5 times (5 min each) and counterstained with aqueous saturated Uranyl Acetate (Polysciences, Inc., Warrington, PA) for 10 minutes and Sato’s Lead Citrate stain for 2 minutes. Control grids were exposed to normal rabbit serum or incubation buffer with the addition of normal goat serum instead of the primary antibody. Grids were viewed on a Zeiss EM 910 electron microscope at 100kV accelerating voltage. Pictures were recorded on Kodak SO-163 electron image films, and processed in a Mohr Pro 8 film/paper processor. Negatives were scanned to produce digital image files.

## RESULTS

### Identification of IP_3_Rs and RyRs in Mouse Lacrimal Gland

**Fig. (1):**
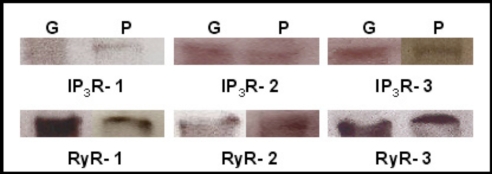
**Identification of IP_3_Rs and RyRs by immunoblotting**. Western blot detection of IP_3_R1-3, RyR1-3 was performed in homogenates of adult lacrimal gland tissue (G) and primary cultures of P10 lacrimal acinar cells (P) yielding bands of approximately 250 and 550-600 kDa, respectively.

Western blot analysis of adult mouse lacrimal gland tissue shows immunoreactivity of each of the three IP_3_Rs and the three RyRs (Fig. **1**). Each IP_3_R isotype was identified using isoform specific antibodies with bands of the expected molecular weight of approximately 250 kDa. Immunoblots also showed expression of the three RyR isotypes with bands of approximately 550-560 kDa.

### Cellular Distribution of IP_3_Rs and RyRs in Mouse Lacrimal Gland

**Fig. (2):**
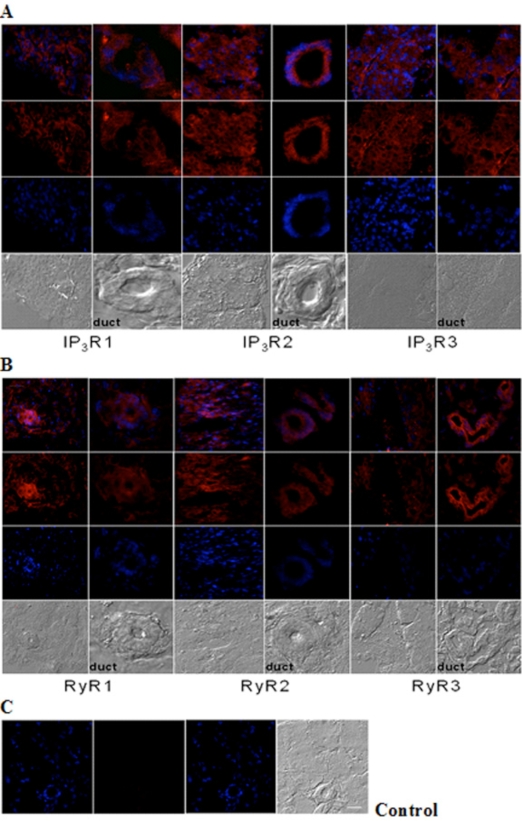
**Immunohistochemical analysis of IP_3_R and RyR distribution in adult mouse lacrimal gland**. Representative images of **(A)** IP_3_R1, IP_3_R2, IP_3_R3 and **(B)** RyR1, RyR2 and RyR3 immunoreactivity. **(C)** Negative control was performed by omission of the primary antibody. Scale bar in **C** for A-C, 25 µm. Red (Alexa Fluor® 594 labeled immunoreactivity) shows receptor signals and the nuclear counterstain (DAPI) is displayed in blue.

Immunohistochemistry analysis of cryosections of adult lacrimal gland tissue shows IP_3_Rs (Fig. **2A**) and RyRs (Fig. **2B**) immunoreactivity. All IP_3_R isoforms were detected in lacrimal acinar cells and in the duct areas, displaying highest levels of IP_3_R type 2 compared to types 1 and 3. And, the staining pattern of IP_3_R types 1 and 3 were similar in both tissue and duct areas. In the case of RYR isotypes, immunoreactivity for all three RyRs was detected as differentially distributed. RyR3 displayed strong immunoreactivity in lacrimal acinar cells of duct areas. In contrast, RyR1 and 2 immunoreactivity were more pronounced in lacimal gland tissue other than ducts.

**Fig. (3):**
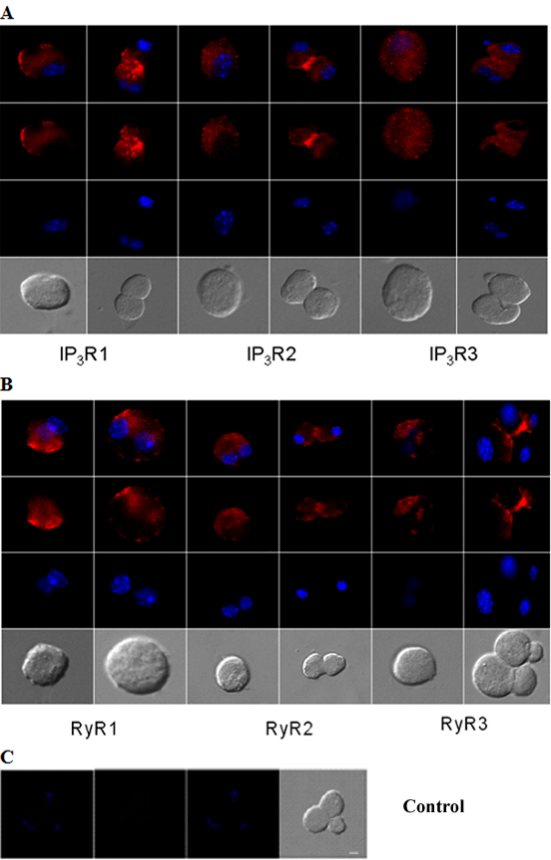
**Immunocytochemical analysis of IP_3_Rs and RyRs distribution in acutely isolated acinar cells of the adult mouse lacrimal gland**. Representative images of **(A)** IP_3_R1, IP_3_R2, IP_3_R3 and **(B)** RyR1, RyR2 and RyR3. **(C)** Omission of primary antibody yielded the negative control. Red (Alexa Fluor® 594 labeled immunoreactivity) shows receptor signals and the nuclear counterstain (DAPI) is displayed in blue. Scale bar in C for A-C, 10 µm.

**Fig. (4):**
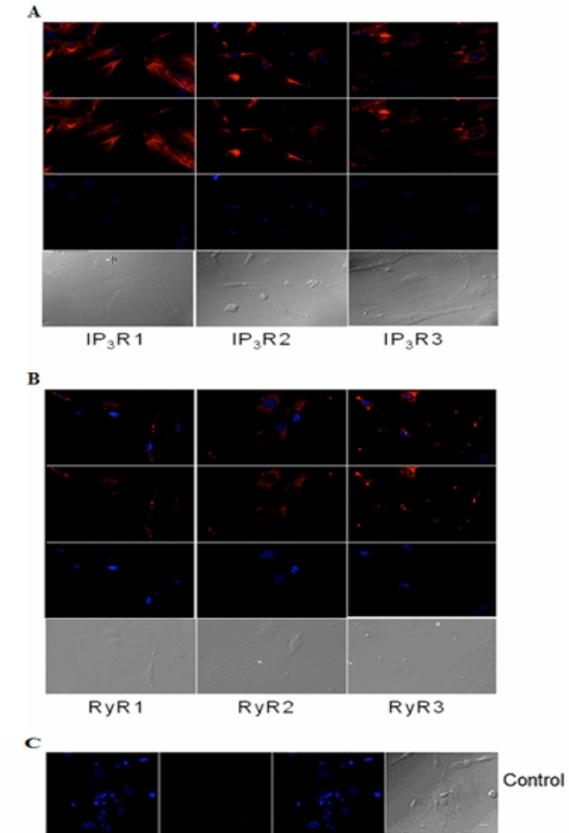
**Immunocytochemical analysis of IP_3_Rs and RyRs distribution in cultured acinar cells of the P10 lacrimal gland after 10 DIV**. Representative images of **(A)** IP_3_R1, IP_3_R2, IP_3_R3 and **(B)** RyR1, RyR2 and RyR3 are shown. **(C)** Primary antibody was omitted for the negative control. Red (Alexa Fluor® 594 labeled immunoreactivity) shows receptor signals and the nuclear counterstain (DAPI) is displayed in blue. Scale bar in **C** for **A-C**, 10 µm.

Similarly, immunocytochemistry of isolated adult acinar cells and primary cultures of isolated P10 lacrimal acinar cells confirmed the expression of the three IP_3_Rs (Figs. **3A**,**4A**). Isolated adult acinar cells (Fig. **3A**) showed expression of IP_3_R1 with intense staining of the ER and the Golgi apparatus. Types 2 and 3 displayed similarly distributed, but less intense staining patterns. Cultures of P10 acinar cells (Fig. **4A**), also showed high levels of IP_3_R1 immunostaining in the ER and nuclear envelope and extending into processes. IP_3_R2 staining was detected as a typical ER and nuclear envelope staining pattern and more concentrated in the cell body than in processes. IP_3_R type 3 immunoreactivity was identified mostly within the cell body rather than in processes, and was most intense in the nuclear envelope.

Compared to immunoreactivity for IP_3_Rs, the distribution of RyR isotypes shows very similar patterns but also extends into sub-plasma lemmal regions of the cell (Figs. **3B**,**4B**). RyR immunoreactivity in isolated adult lacrimal acinar cells (Fig. **3B**) and primary cultures of P10 lacrimal acinar cells (Fig. **4B**) is found concentrated in the cell body. Immunoreactivity for RyR2 and RyR3 is located in the nuclear membrane and is particularly pronounced for RyR3. While RyR1 immunoreactivity is more uniformly distributed throughout the cell body.

### EM Analysis of Intracellular Calcium Channel Immunoreactivity in Mouse Lacrimal Gland

**Fig. (5):**
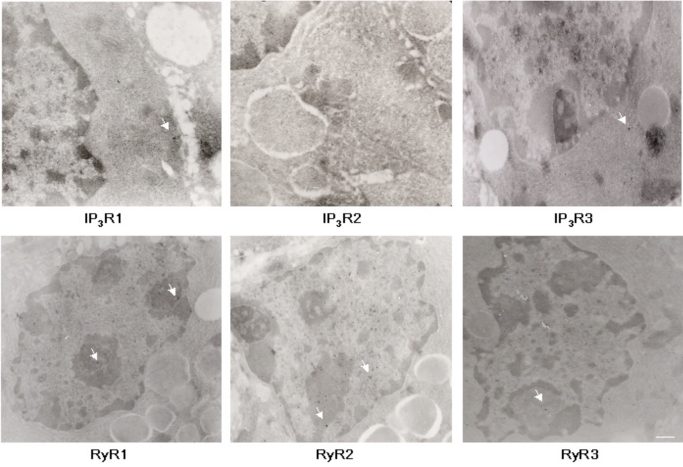
**EM analysis of IP_3_R and RyR immunoreactivity in adult lacrimal gland sections**. Representative images of IP_3_R1, IP_3_R2, IP_3_R3, RyR1, RyR2, RyR3 staining. Arrows indicate 15 nm immunogold label in different subcellular compartments. Scale bar, 100nm (RyR1, RyR2, RyR3, IP_3_R3, and 160nm (IP_3_R1 and IP_3_R2).

**Fig. (6):**
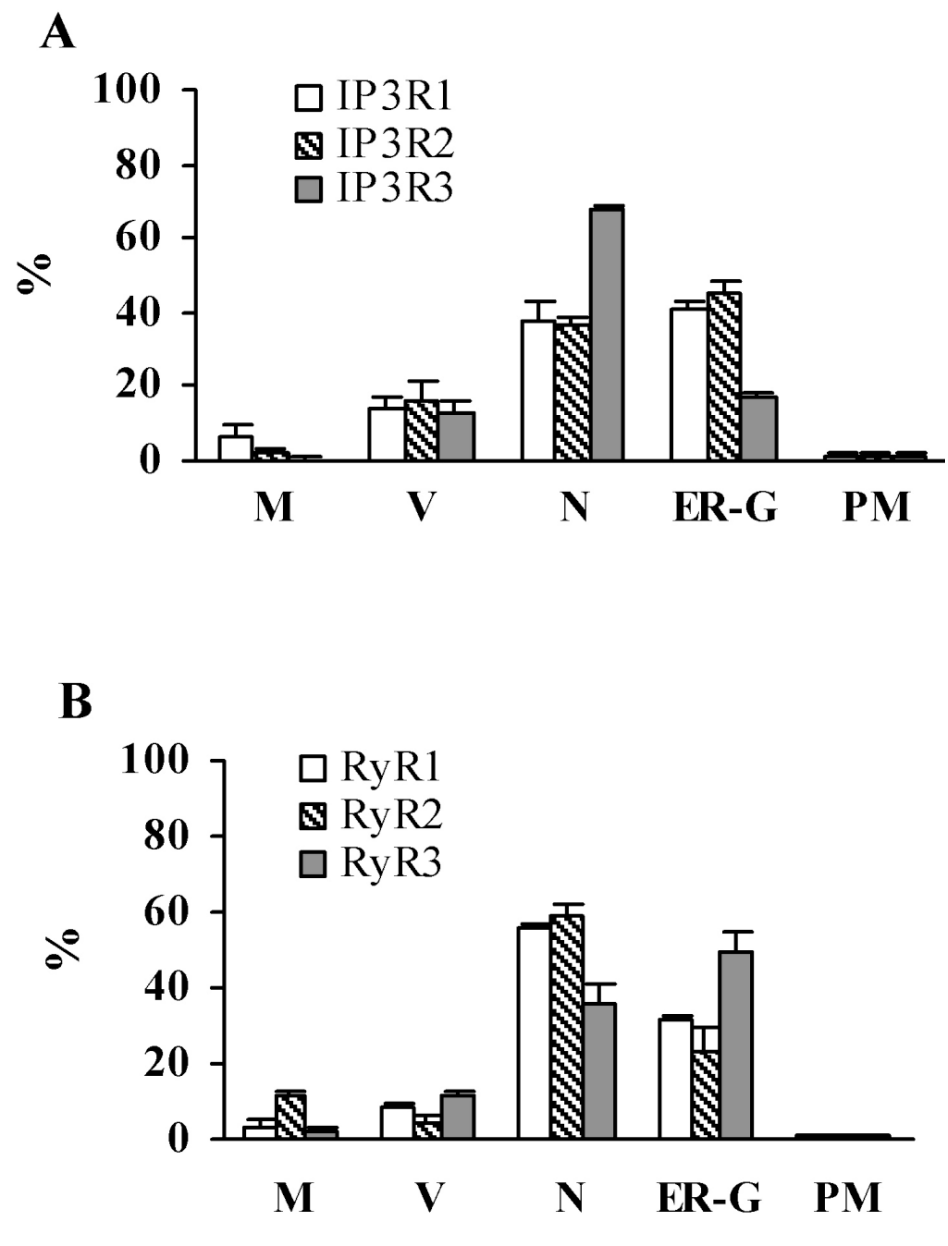
**EM semi-quantitative analysis of ultrastructural immunoreactivity in adult mouse lacrimal gland tissue**. Graph **A** shows immunoreactivity for IP_3_R1, 2, and 3 and graph **B**; for RyR1, 2, and 3. Immunoreactivity in Mitochondria (M), Vesicles (V), Nucleus (N) ER-Golgi Apparatus (ER-G), and Plasma Membrane (PM) displayed along *x* axis. Percentage of total particles (minus controls) indicated along *Y* axis. The subcellular regions most highly associated with IP_3_R1 and 2 immunoreactivity are the nucleus, ER and Golgi apparatus, while the nuclear structures are more highly associated with IP_3_R3. Analysis shows RyR1 and 2 to be more highly expressed in nuclear structures and ER-Golgi apparatus, while RyR3 is expressed in ER-Golgi with less immunoreactivity in the nucleus.

In order to semi-quantitatively determine immunoreactivity for intracellular calcium channels, ultrastructural immuno-EM analyses were performed on adult mouse lacrimal gland sections (Fig. **5**). The subcellular structures most highly associated with IP_3_R1 and 2 immunoreactivity are the nucleus, ER and Golgi apparatus, while nuclear structures are most highly associated with IP_3_R3 immunoreactivity (Fig. **6**). Interestingly, immunoreactivity for IP_3_Rs was also found on secretory vesicles. EM analysis of RyR isotype immunoreactivity suggests higher expression of RyR 1 and 2 in nuclear structures and the ER-Golgi apparatus area. Like IP_3_R, RyRs are also expressed on secretory vesicles but to a lesser extent (Fig. **6**).

## DISCUSSION

Using immunofluorescence microscopy and western blot analysis, we identified the presence and cellular distribution of IP_3_R and RyR intracellular calcium channels and their isotypes in P10 primary cultures and adult lacrimal gland acinar cells. Both analyses identified each of the IP_3_R and RyR isotypes in the mouse lacrimal gland. Light microscopy analysis shows differential subcellular distribution for each of the calcium channel isotypes. Nuclear envelope, Golgi apparatus, the endoplasmic reticulum and its associated intracellular compartments, represent the areas of highest levels of immunoreactivity within the cell for all channels. Ultrastructural detection of immunoreactivity using electron microscopy of adult lacrimal gland sections confirmed the predominant expression of these channels on intracellular membranes seen at the light microscope level.

The results complement pharmacological data that correlate the activation of intracellular calcium channels with the secretory activity of the lacrimal gland. In fact, physiological evidence argues for the existence of distinct pathways utilizing IP_3_R and RyR responding to cholinergic or β-adrenergic stimulation respectively [6-9, 15]. The presence of both types of receptors indicates that these calcium channels potentially contribute to secretion after distinct neuronal stimuli.

The differential intracellular distribution of IP_3_Rs and RyRs determined in the present study and their respective distinct affinities for ligands [10,22] lead to a potential functional role of these calcium channels in the specificity and control of intracellular Ca^2+^ transients. For example, the observed pronounced immunoreactivity for IP_3_R3 in the nuclear envelope parallel that found in functionally related acinar cells of the pancreas and salivary glands [24]. Nuclear localization indicates a potential role for IP_3_R3 in nucleoplasmic Ca^2+^ regulation and related gene expression. In addition, the present study provides evidence for the presence and distribution of IP_3_Rs and RyRs in cultured P10 lacrimal gland cells, an *in vitro* model for measuring lacrimal acinar cell function.

In conclusion, our study shows that both adult and postnatal developing acinar cells from mouse lacrimal gland express all isotypes of IP_3_R and RyR in the endoplasmic reticulum and its related membrane structures such as nuclear envelope, Golgi apparatus and secretory vesicles. This provides a basis for the further investigation of calcium signaling as a therapeutic target for treatment of ocular diseases affecting tear production.
